# Intra-abdominal infection after tumor surgery: tigecycline combined with β-lactam antibiotics versus tigecycline alone

**DOI:** 10.1186/s12885-023-11169-7

**Published:** 2023-07-20

**Authors:** Xinfeng Cai, Hongxia Yan, Wenjun Zhang, Wei Zhao, Lei Zhang, Xu Wang, Xinjing Wu, Zhiying Hao, Jinlin Guo

**Affiliations:** 1 Department of Pharmacy, Shanxi Province Cancer Hospital/Shanxi Hospital Affiliated to Cancer Hospital, Chinese Academy of Medical Sciences/Cancer Hospital Affiliated to Shanxi Medical University, Zhigongxincun Street 3#, 030012 Taiyuan, Shanxi P. R. China; 2 Department of Pharmacy, Second Hospital of Shanxi Medical University, Taiyuan, Shanxi P. R. China; 3 Department of Pharmacy, First Hospital of Shanxi Medical University, Taiyuan, Shanxi P. R. China; 4 Department of Pharmacy, Shanxi Provincial People’s Hospital, Shuangtasi Street 59#, 030001 Taiyuan, Shanxi P. R. China; 5 Department of Literature search, Shanxi Research Center for Information and Strategy of Science and Technology, Taiyuan, Shanxi P. R. China; 6 Department of Pharmacy, Yuncheng Central Hospital, Taiyuan, Shanxi P. R. China

**Keywords:** Tigecycline, β-lactam antibiotics, Postoperative intra-abdominal infection, Monotherapy, Carbapenem-resistant organisms

## Abstract

**Backgrounds:**

Tigecycline has a broad spectrum of antimicrobial activity and has been approved for the treatment of complicated intra-abdominal infections. However, it is debatable whether tigecycline should be used alone or in combination. This study aimed to investigate whether tigecycline plus β-lactam antibiotics (combination therapy [CT] group) are superior to tigecycline alone (monotherapy [MT] group) in non-critically ill intra-abdominal infection patients after tumor surgery.

**Methods:**

This was a multicenter, retrospective cohort study. The primary outcome was mortality during the hospital stay. Secondary outcomes were clinical success rate, microbial eradication rate, relapse rate within one week, course of treatment, and adverse effects. Propensity score matching (PSM) was used to adjust the degree of infection before medication between the MT and CT groups. Univariate comparisons were performed using the chi-squared test for qualitative variables and Student’s t-test or the Mann-Whitney U-test for continuous variables, as appropriate. Multivariate logistic regression analysis was performed to examine the relationship between antimicrobial treatments and mortality during hospitalization. The paired samples Wilcoxon test was used to compare the parameters before and after medication.

**Results:**

In total, 291 patients were included in the final analysis: 128 in MT group and 163 in CT group. Mortality rate was 6.25% in the MT group and 6.13% in the CT group (*P* = 0.97). Multivariate logistic regression model showed that carbapenem-resistant organisms (OR: 4.35, 95% CI: 2.36 ~ 61.70) and age > 65 (OR: 1.32, 95% CI:1.19 ~ 3.01) were independent risk factors for death. CT group had a shorter defervescence time (*P* < 0.05), with less likelihood of relapse (*P* < 0.05) but had a more significant effect on activated partial thromboplastin and prothrombin time.

**Conclusions:**

Tigecycline plus β-lactam wasn’t superior to tigecycline monotherapy for the treatment of non-critically ill patients with intra-abdominal infection. But for advanced age patients with cancer, tigecycline combination therapy maybe a better choice in terms of mortality.

**Supplementary Information:**

The online version contains supplementary material available at 10.1186/s12885-023-11169-7.

## Background

Intra-abdominal infection (IAI) is a common complication of abdominal surgeries. Postoperative intra-abdominal infection (PIAI) is an important type of IAI, accounting for approximately 8.5% of total IAIs, and its mortality rate is as high as 22%~55%[1]. PIAI refers to the clinical manifestations of IAI within 30 days of surgery, with laboratory tests and imaging confirming IAI or drainage fluid confirming the presence of an intra-abdominal abscess [1]. Appropriate empirical antimicrobial treatment can increase the success rate of clinical treatment, reduce hospital stay and hospitalization costs, and minimize antimicrobial resistance caused by selective pressure [2].

Multidrug-resistant (MDR) bacteria, such as methicillin-resistant *Staphylococcus aureus*, extended-spectrum beta-lactamase-producing Enterobacteriaceae, carbapenemase-producing *Klebsiella pneumoniae*, and carbapenem-resistant *Acinetobacter baumannii* (CRAB), are common in intra-abdominal infections [3–5]. Cancer is the risk factor for MDR bacteria infection [6]. Therefore, it is challenging for physicians to choose an appropriate anti-infection regimen for IAI after tumor surgery.

In China and the US, tigecycline is approved for the treatment of complicated intra-abdominal infections, complicated skin and skin tissue infections, and community-acquired bacterial pneumonia. It is a minocycline derivative with antimicrobial activity against Gram-positive and Gram-negative bacteria, anaerobes, and atypical pathogens. Tigecycline is often used in combination with other antibiotics, such as β-lactam antibiotics, carbapenems, and aminoglycosides, owing to its heterogeneous resistance [7, 8]. The combination of tigecycline with β-lactam antibiotics has shown a good synergistic effect [9, 10], and tigecycline plus cefoperazone/sulbactam is the first-line treatment for CRAB in China. However, tigecycline has a higher concentration in the bile, gall bladder and colon [11, 12], which means that tigecycline is more effective against IAI compared to other site of infections. Furthermore, its unique pharmacological mechanism provides good antibacterial activity against various pathogenic bacteria that cause complicated intra-abdominal infections, especially MDR bacteria [13, 14]. Some studies suggested that tigecycline alone is not inferior to tigecycline-based combination regimens [15–19], while whether tigecycline should be used alone or in combination in PIAI cancer patients was remained unknown. This study aimed to investigate whether tigecycline combined with β-lactam antibiotics (combination therapy group, CT group) is superior to tigecycline alone (monotherapy group, MT group) in intra-abdominal infection after tumor surgery.

## Methods

This was a five-center (in China), retrospective cohort study. The study design was based on a comparison of outcomes between two groups of patients with PIAIs. The primary outcome in this study was mortality during the hospital stay. The secondary outcomes were clinical success rate, microbial eradication rate, relapse rate within one week, course of treatment, and adverse effects.

### Cohort description

**Fig. 1:**
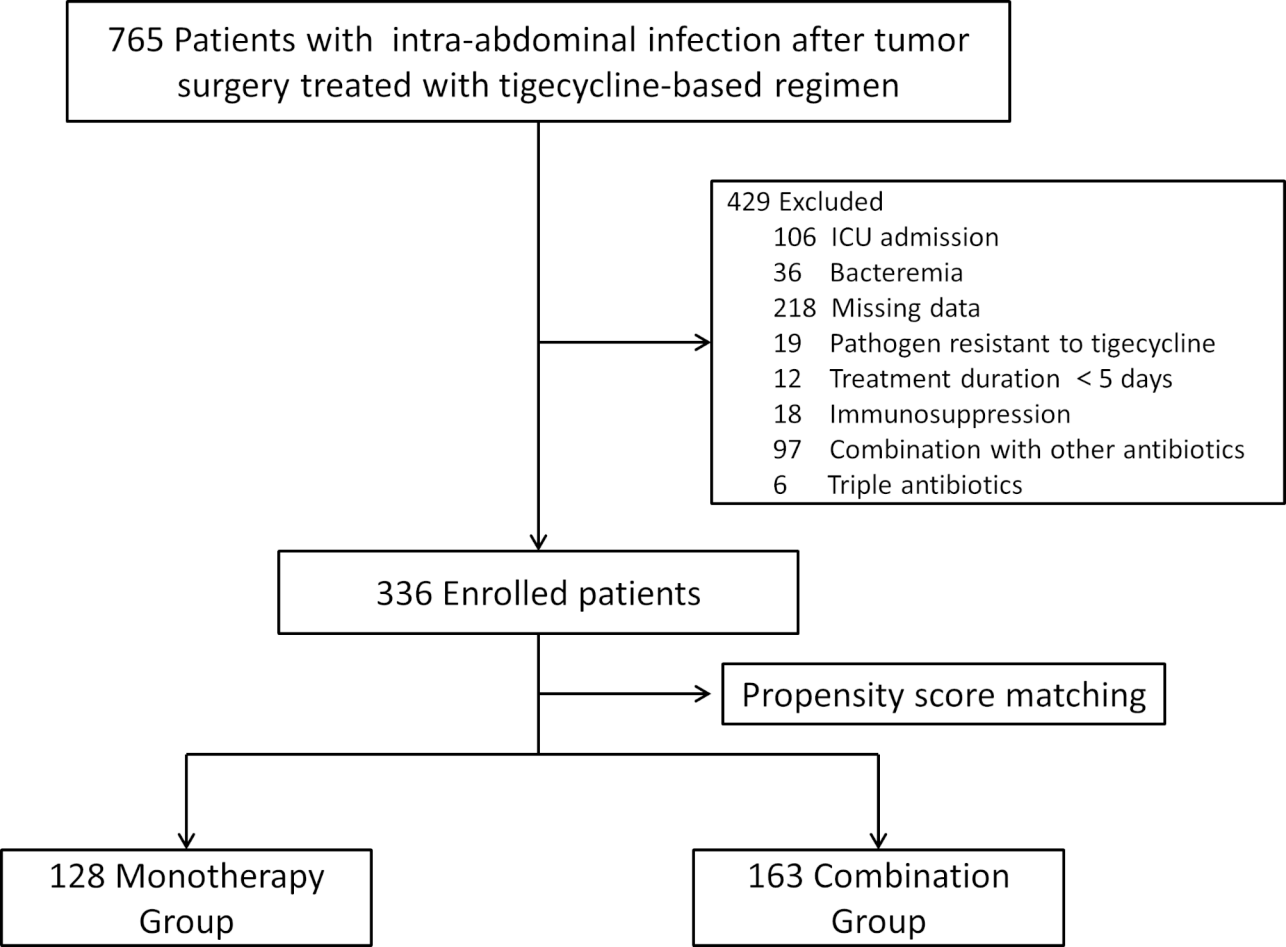
Flowchart of patient enrollment

The sample groups for this cohort study were derived from individuals registered at the gastrointestinal surgery departments of Shanxi Provincial People’s Hospital, First Hospital of Shanxi Medical University, Second Hospital of Shanxi Medical University, Yuncheng Central Hospital and Shanxi Cancer Hospital; each of these hospitals was a tertiary hospital with over 2,200 inpatient beds. We retrospectively reviewed the clinical records of adult cancer patients (age > 18 years) with PIAI who were treated with tigecycline or tigecycline in combination with β-lactam antibiotics between January 2018 and November 2022. The study protocol was approved by the Ethics Committee of Shanxi Provincial People’s Hospital (2022 − 281). The need to obtain written informed consent was waived due to the retrospective nature of the study. A total of 336 participants who met the inclusion criteria were enrolled in the study (Fig. 1).

### Definitions

Intra-abdominal infection must meet the following criteria: presence of organisms isolated from abdominal drainage, body temperature > 37.5 ^o^C, and abdominal pain. Clinical success was defined as the absence of any sign of infection at the end of treatment, normal temperature, no indication for continuation of antibiotics treatment or a second operation, and no relapse within one week. Microbial eradication was defined as the absence of pathogens in the culture of specimens collected from the original site. Relapse was defined as the recurrence of abdominal pain within one week of the end of antibiotics treatment, with or without unexplained fever, increase in white blood cells, C-reactive protein, or procalcitonin (PCT), new radiographic findings (abscess or other infectious manifestations), and cannot be explained by infection at other sites.

### Inclusion definition for the Monotherapy and Combination Groups

The MT group was defined as patients with PIAI treated with tigecycline alone: a loading dose of 100 mg, followed by 50 mg twice daily. The CT group was defined as patients with PIAI treated with tigecycline in combination with β-lactam antibiotics (ceftazidime, piperacillin/tazobactam, cefoperazone/sulbactam, ceftriaxone or cefoxitin); the dose of tigecycline in the CT group was the same as that in the MT group. The inclusion criteria were as follows: patients who had undergone abdominal surgery and had been diagnosed with IAI, male or female, older than 18 years, and treated with tigecycline or tigecycline in combination with β-lactam antibiotics. The exclusion criteria were as follows: missing data; use of tigecycline within the last month; the pathogen was resistant to tigecycline; bacteremia; intensive care unit admission; course of treatment was less than five days; use of immunosuppressants or immunodeficiencies; mechanical ventilation; renal replacement therapy; combination with other antibiotics; three antibiotics were used at the same time; and liver failure.

The patients’ baseline data (age, sex, underlying diseases, causative pathogens, course of treatment, type of surgery, and laboratory tests) were obtained from the Hospital Information System. Laboratory tests between 48 h before tigecycline administration and within 48 h after tigecycline discontinuation were recorded.

### Statistical analysis

Propensity score matching (PSM) was used to adjust the degree of infection before medication (age, PCT) between the MT and CT groups. The Shapiro-Wilk test was used to test for normality. Univariate comparisons were performed using the chi-squared test for qualitative variables and Student’s t-test or the Mann-Whitney U-test for continuous variables, as appropriate. The paired samples Wilcoxon test was used to compare the parameters before and after medication. Conditional logistic regression model was used to investigate the correlation between the parameters and mortality. The significance level was set at *P* < 0.05. All statistical analyses were calculated using Extreme Smart Analysis platform. Retrieved from https://www.xsmartanalysis.com.

## Results

After applying the inclusion and exclusion criteria, the study cohort included 336 patients. Statistical analysis revealed significant differences in age and PCT levels between the MT and CT groups. PSM was used to match the two groups to eliminate baseline differences. After PSM, 128 and 163 patients were included in the MT and CT groups, respectively. The baseline clinical characteristics are presented in Table 1.

**Table 1:** Features of patients with post-surgical intra-abdominal infections

Parameter	MT Group	CT Group
Number of patients	128	163
Sex (n, %)		
Male	98 (76.56)	104 (63.80)
Female	30 (23.44)	45 (36.20)
Age (years)	56.10 ± 7.67	56.30 ± 7.43
Underlying diseases, n (%)		
Diabetes	10 (7.81)	13 (7.98)
CKD	2 (1.56)	2 (1.23)
CHF	0	3 (1.84)
COPD	10 (7.81)	5 (3.07)
Site of tumor, n (%)		
Liver	24 (18.75)	18 (11.04)
Biliary tract	38 (29.69)	39 (23.93)
Gastrointestinal tract	50 (39.06)	94 (57.67)
Pancreas	16 (12.50)	12 (7.36)
Responsible pathogens, n (%)		
*Enterococcus.* spp.	11 (8.59)	29 (17.79)
*Escherichia coli* (total)	38 (29.69)	60 (36.81)
*Escherichia coli* (CRO)	8 (6.25)	6 (3.68)
*Klebsiella* spp. (total)	26 (20.31)	32 (19.63)
*Klebsiella* spp. (CRO)	6 (4.69)	10 (6.13)
*Enterobacter cloacae* (total)	15 (11.72)	9 (5.52)
*Enterobacter cloacae* (CRO)	4 (3.13)	5 (3.07)
*Acinetobacter baumannii* (CRO)	10 (7.81)	18 (11.04)
other bacteria	28 (21.88)	15 (9.20)
Antibiotic combination, n (%)		
Ceftazidime	N/A	16 (9.82)
Piperacillin/tazobactam	N/A	48 (29.45)
Cefoperazone/sulbactam	N/A	89 (54.60)
Ceftriaxone	N/A	4 (2.45)
Cefoxitin	N/A	6 (3.68)
Parameters before tigecycline medication		
Temperature (℃)	38.31 ± 0.47	38.42 ± 0.36
CRP (mg/L)	77.14 ± 20.18	81.25 ± 36.71
PCT (ng/mL)	2.50 ± 1.06	2.48 ± 0.64
White Blood Cell (*10^9^/L)	18.12 ± 3.77	21.69 ± 2.60
Hospital length of stay (days)	13.43 ± 5.12	15.76 ± 8.25

MT Group: tigecycline monotherapy; CT Group: tigecycline plus β-lactam antibiotics; CRO, Carbapenem-resistant organisms protein; CRP, C-reactive protein; PCT: Procalcitonin; CHF: Congestive heart failure; CKD: Chronic kidney disease; COPD: Chronic obstructive pulmonary disease; CRO: Carbapenem-resistant organisms

The comparisons between the two groups are presented in Table 2. There was no significant difference in mortality during hospitalization between the two groups (6.25% vs., 6.13%, P = 0.97). Clinical efficacy was 80.47% (103/128) in the MT group and 86.50% (141/163) in the CT group (P = 0.17). Microbial eradication rates were 92.19% (118/128) in the MT group and 94.48% (154/163) in the CT group, respectively (P = 0.43). In addition, there was no significant difference in duration of microbial eradication (5.12 ± 1.38 vs. 4.93 ± 1.51, P = 0.78) and duration of clinical success (7 [6, 9] vs. 7 [6, 9], P = 0.76). However, the MT group needed a longer time for the temperature to return to normal (4.67 ± 0.32 vs. 3.89 ± 0.45, P < 0.05). In addition, the MT group was more likely to relapse within a week (14.06% vs. 6.13%, P < 0.05).

**Table 2:** Efficacy comparison between the two groups

Parameter	MT group	CT group	*P*
Mortality during hospitalization, n (%)	8 (6.25)	10 (6.13)	0.97
Clinical success, n (%)	103 (80.47)	141 (86.50)	0.17
Microbial eradication, n (%)	118 (92.19)	154 (94.48)	0.43
Defervescence time (days)	4.67 ± 0.32	3.89 ± 0.45	< 0.05^*^
Duration of microbial eradication (days)	5.12 ± 1.38	4.93 ± 1.51	0.78
Duration of clinical success (days)	7 (6, 9)	7 (6, 9)	0.76
Relapse within one week, n (%)	18 (14.06)	10 (6.13)	< 0.05^*^

* Statistically significant

Multivariate analysis suggested that treatment regimen (OR: 1.57, 95% CI: 0.97–4.03) was not associated with mortality, but with age > 65 (OR: 1.32, 95% CI:1.19 ~ 3.01) and carbapenem-resistant organisms (CRO) [OR: 4.35, 95% CI: 2.36–61.70] (Table 3).

**Table 3:** Factors influencing mortality during hospitalization for patients with postoperative intra-abdominal infection

Parameter	OR	95% Cl	*P* ^a^
CRO	4.35	2.36 ~ 61.70	< 0.01^*^
Age > 65	1.32	1.19 ~ 3.01	0.03^*^
Monotherapy/combination	1.57	0.97 ~ 4.03	0.06

^a^: Multivariate analysis with conditional logistic regression model

CRO: Carbapenem-resistant organisms

Furthermore, we compared changes in laboratory test results before and after tigecycline-based medication between the groups (Table 4). Coagulation function (activated partial thromboplastin time, APTT; prothrombin time, PT; fibrinogen, FIB) was significantly lower after tigecycline-based treatment than that before the treatment (*P* < 0.05) in each group. There was no difference in alanine aminotransferase (ALT) and aspartate aminotransferase (AST) levels before and after medication for both groups. Total bilirubin (Tbil) in the MT group was significantly elevated after the treatment (14.93 ± 46.23 vs. 18.94 ± 40.55, *P* <0.05). The hemoglobin levels in the CT group were significantly reduced after treatment (100 (89, 112.75) vs. 96 (86, 112), *P* < 0.05). There was no significant change in platelet levels in both groups before and after treatment.

**Table 4:** Adverse reactions between the MT and CT groups

Parameter	Group	Before medication	After medication	*P*
APTT (seconds)	MT	30.30 ± 6.05	32.90 ± 7.47^&^	< 0.01^*^
	CT	30.60 ± 6.19	35.20 ± 8.20	< 0.01^*^
PT (seconds)	MT	13.80 ± 2.32	14.30 ± 2.75^&^	< 0.01^*^
	CT	14.35 ± 2.36	15.20 ± 4.28	< 0.01^*^
FIB (g/L)	MT	4.14 ± 1.59	2.82 ± 1.43	< 0.01^*^
	CT	4.05 ± 1.69	2.59 ± 1.34	< 0.01^*^
Tbil (umol/L)	MT	14.93 ± 46.23^#^	18.94 ± 40.55	< 0.05^*^
	CT	20.23 ± 70.79	28.06 ± 77.19	0.32
ALT (U/L)	MT	41.94 ± 55.36	41.70 ± 275.17	0.78
	CT	41.97 ± 74.09	35.13 ± 90.59	0.09
AST (U/L)	MT	39.98 ± 49.74	34.90 ± 379.36^&^	0.69
	CT	44.63 ± 100.19	41.23 ± 218.97	0.63
Hemoglobin (g/L)	MT	99 (84, 111)	98 (85, 108)	0.28
	CT	100 (89, 112.75)	96 (86, 112)	< 0.05^*^
Platelet (*10^9^/L)	MT	196.5 (123, 301.5)	210 (134, 300)	0.82
	CT	216 (98, 307)	214 (117, 306)	0.63

MT: tigecycline monotherapy; CT: tigecycline plus β-lactam antibiotics; APTT, activated partial thromboplastin time; PT, prothrombin time; FIB, fibrinogen; Tbil, total bilirubin; ALT, alanine aminotransferase; AST, aspartate aminotransferase; *, statistically significant; ^#^, statistically significant between the monotherapy and combination groups in levels of total bilirubin before tigecycline medication; ^&^, statistically significant between monotherapy and combination groups after tigecycline medication

To compare the adverse effects after medication between the MT and CT groups, we first compared the parameters between the two groups before treatment to eliminate baseline differences and found that only total bilirubin was significantly different between the two groups before treatment. Afterward, we compared other parameters between the MT and CT groups after medication and found that only APTT, PT, and AST were statistically different between the two groups at the end of treatment.

## Discussion

Several clinical studies have documented the effects of tigecycline as a single agent or in combination with other antimicrobials, and only three studies involved intra-abdominal infection [15–18, 20–22]. Cancer is the risk factor of MDR intra-abdominal infection. For cancer patients with IAI, whether tigecycline should be used alone or combination is still unknown. On searching PubMed and Web of Science, we believe that this is the first study to compare the efficacy and adverse effects of tigecycline monotherapy and tigecycline in combination with β-lactam antibiotics in IAI patients after tumor surgery.

It is debatable whether tigecycline should be used alone or in combination [23, 24]. Many studies have supported a combination regimen for hospital-acquired pneumonia [20, 24, 25]. For intra-abdominal infections, tigecycline alone has shown a success rate of up to 90%, with the in-hospital mortality and bacterial eradication rates not significantly different [16, 17]. In the present study, the addition of β-lactam antibiotics to tigecycline in PIAI patients was not associated with better clinical outcomes, including mortality during hospitalization, clinical success rate, microbial eradication rate, duration of microbial eradication, defervescence time, and duration of clinical success. We chose mortality during the hospital stay as the primary outcome instead of 28 days because the patients enrolled in this study had milder disease. Furthermore, at 28 days, mortality may have been influenced by underlying comorbidities.

Our findings were consistent with those of previous studies [16, 17, 26]. We observed that tigecycline plus β-lactam was not superior to tigecycline monotherapy for the treatment of non-critically ill patients with IAI after tumor surgery. We believe that this was due to the high concentration of tigecycline in the abdomen and its broad antimicrobial spectrum [12]. Therefore, tigecycline is effective in the treatment of mild abdominal infections, even when used alone. However, tigecycline concentration in the blood is very low, and therefore, should not be used for bacteremia [27]. As a result, we excluded patients with bacteremia and ICU admission, and our findings were limited to non-critically ill patients.

Although there was no difference in mortality during hospitalization between the two groups, the defervescence time of patients in the CT group was significantly lower than that of patients in the MT group. Tigecycline is a bacteriostatic agent, whereas β-lactam antibiotics are bactericidal agents; thus, more time is needed to lower the body temperature for tigecycline monotherapy [28]. The results of bacterial eradication rates may seem contradictory to relapse rates; however, given that the frequency of abdominal drainage culture performed for each patient was different and that there were many factors affecting bacterial culture, false negatives were likely to exist. Therefore, we believe that the results of relapse rates may be more reliable than those of bacterial eradication rates.

Multivariate analysis with a logistic regression model showed that mortality was not associated with tigecycline use alone, but with CRO and advanced age. First, tigecycline requires a high dose of CRO (200 mg loading dose, followed by 100 mg every 12 h)[8, 29, 30]; however, patients enrolled in this study were treated with a standard dose. Second, low doses of tigecycline have a higher risk of selecting drug-resistant isolates [23]. Third, advanced age and cancer were risk factors of MDR infection, which was related to treatment failure [6, 31]. Furthermore, superinfections from CRO secondary to tigecycline resistance were more common during monotherapy than during combination therapy [32]. Therefore, we suggest that even for non critical ill postoperative intra-abdominal infections, physicians should choose tigecycline combination therapy to reduce mortality for advanced age patients with cancer.

According to the manufacturer’s instructions, tigecycline may cause coagulopathy [11]. The effect of tigecycline on coagulation function mainly manifests as a prolongation of PT and APTT and a decrease in FIB [33–36], and our study supports this hypothesis. Antibiotics are generally associated with coagulation disorders, as they reduce the microflora of the colon and distal ileum, which synthesize vitamin K2[37, 38]. However, the mechanism underlying this effect remains unclear. Comparison of coagulation function between the two groups showed that the CT group had a more pronounced effect on APTT and PT than did the MT group. This may be because both tigecycline and β-lactam antibiotics may cause coagulopathy, and the combination of these two drugs exacerbates this adverse effect. Therefore, patients at risk of bleeding should be closely monitored during tigecycline treatment. Although tigecycline-based regimens have a significant effect on coagulation, patients usually recover spontaneously after drug discontinuation [36].

Regarding the effect on liver function, although AST was higher in the CT group, we did not think this result was meaningful considering that many drugs affect aminotransferases, and we cannot completely rule out the effect of other drugs on aminotransferases.

There are some limitations of this study. First, this was a retrospective study and was thus susceptible to selection bias. Although we have used PSM to reduce bias, some potential sources of biases may be neglected. Second, adequate drainage is critical for treatment of PIAI; however, we cannot confirm that each patient received adequate drainage. Third, the sample size is relatively small.

## Conclusions

In conclusion, tigecycline alone or in combination with β-lactam antibiotics was not associated with mortality during hospitalization, clinical success, or microbial clearance. CRO infection and advanced age were independent risk factors for death. Tigecycline in combination with β-lactam antibiotics has a shorter defervescence time and is less likely to be associated with a relapse; however, it has a more significant effect on coagulation. But for advanced age patients with cancer, tigecycline combination therapy maybe a better choice in terms of mortality.

## Electronic supplementary material

Below is the link to the electronic supplementary material.

Supplementary Material 1

## Data Availability

The data that support the findings of this study are available from the corresponding author (Professor Jinlin Guo) upon reasonable request.

## List of abbreviations

CT group: combination therapy group
MT group: monotherapy group
IAI: Intra-abdominal infection
PIAI: Postoperative intra-abdominal infection
MDR: Multidrug-resistant
CRAB: Carbapenem-resistant *Acinetobacter baumannii*
PCT: procalcitonin
PSM: Propensity score matching
OR: Odds ratio
CI: Confidence Interval
CRO: carbapenem-resistant organisms
APTT: activated partial thromboplastin time
PT: prothrombin time
FIB: fibrinogen
ALT: alanine aminotransferase
AST: aspartate aminotransferase
Tbil: Total bilirubin
