# Supplementary material for: Intra-abdominal infection after tumor surgery: tigecycline combined with β-lactam antibiotics versus tigecycline alone
Source: BMC Cancer. 2023 Jul 20;23:682. doi: 10.1186/s12885-023-11169-7 (PMC10357740; doi:10.1186/s12885-023-11169-7)
Supplement: Supplementary file 1 — Supplementary Material 1 [file 12885_2023_11169_MOESM1_ESM.docx]

**Table 1 Proposed search terms**

| Search | Query | Results |
| --- | --- | --- |
| #1 | Search: (tigecycline[Title/Abstract]) AND (cancer[Title/Abstract]) | 60 |
| #2 | Search: (tigecycline[Title/Abstract]) AND (tumor[Title/Abstract]) | 25 |
| #3 | Search: "tigecycline"[Title/Abstract] AND "cancer"[Title/Abstract] AND "monotherapy"[Title/Abstract] | 3 |
| #4 | Search: "tigecycline"[Title/Abstract] AND "tumor"[Title/Abstract] AND "monotherapy"[Title/Abstract] | 2 |
| #5 | Search: "tigecycline"[Title/Abstract] AND "monotherapy"[Title/Abstract] | 180 |
| #6 | Search: "tigecycline"[Title/Abstract] AND "alone"[Title/Abstract] | 219 |
| #7 | Search: "tigecycline"[Title/Abstract] AND "alone"[Title/Abstract] AND "cancer"[Title/Abstract] | 10 |
| #8 | Search: "tigecycline"[Title/Abstract] AND "alone"[Title/Abstract] AND "tumor"[Title/Abstract] | 4 |

Search was conducted on 15, Dec, 2021.

**Table 2 Update search results**

| Search | Query | Results |
| --- | --- | --- |
| #1 | Search: (tigecycline[Title/Abstract]) AND (cancer[Title/Abstract]) | 71 |
| #2 | Search: (tigecycline[Title/Abstract]) AND (tumor[Title/Abstract]) | 33 |
| #3 | Search: "tigecycline"[Title/Abstract] AND "cancer"[Title/Abstract] AND "monotherapy"[Title/Abstract] | 3 |
| #4 | Search: "tigecycline"[Title/Abstract] AND "tumor"[Title/Abstract] AND "monotherapy"[Title/Abstract] | 2 |
| #5 | Search: "tigecycline"[Title/Abstract] AND "monotherapy"[Title/Abstract] | 188 |
| #6 | Search: "tigecycline"[Title/Abstract] AND "alone"[Title/Abstract] | 233 |
| #7 | Search: "tigecycline"[Title/Abstract] AND "alone"[Title/Abstract] AND "cancer"[Title/Abstract] | 11 |
| #8 | Search: "tigecycline"[Title/Abstract] AND "alone"[Title/Abstract] AND "tumor"[Title/Abstract] | 5 |

Search was conducted on 2, Jan, 2023.
